# Supplementary material for: A UMLS-based spell checker for natural language processing in vaccine safety
Source: BMC Med Inform Decis Mak. 2007 Feb 12;7:3. doi: 10.1186/1472-6947-7-3 (PMC1805499; doi:10.1186/1472-6947-7-3)
Supplement: Additional file 13 — RAP application directory. Sets up directory for RAP (RDF application for PHP) source code [file 1472-6947-7-3-S13.gz › rap/doc/phpdoc/utility/Object.html]

xml version="1.0" encoding="iso-8859-1"?


Docs For Class Object


## Class Object

Description

Description |
Descendents
| Methods (details)

An abstract object.

Root object with some general methods, that should be overloaded.

History:

- 09-10-2002 : First version of this class.

- abstract:
- author: Chris Bizer <chris@bizer.de>
- version: V0.9.1

Located in /util/Object.php (line 24)

Direct descendents

Description |
Descendents
| Methods (details)

| Class | Description |
| --- | --- |
| DbStore | DbStore is a persistent store of RDF data using relational database technology. |
| Model | Abstract superclass of MemModel and DbModel. A model is a programming interface to an RDF graph. |
| Node | An abstract RDF node. |
| Statement | An RDF statement. |
| RdqlEngine | Some general methods common for RdqlMemEngine and RdqlDbEngine |
| RdqlParser | This class contains methods for parsing an Rdql query string into PHP variables. |
| RdqlResultIterator | Iterator for traversing Rdql results. |
| GRDDLParser | A GRDDLParser. |
| N3Parser | PHP Notation3 Parser |
| N3Serializer | An abstract object. |
| NTripleSerializer | PHP N-Triple Serializer |
| RdfParser | An RDF paser. |
| RdfSerializer | An RDF seralizer. |
| FindIterator | Iterator for traversing statements matching a searchpattern. |
| RDFUtil | Useful utility methods. |
| StatementIterator | Iterator for traversing models. |

Method Summary

Description |
Descendents |
Methods (details)

string
toString
()

Methods

Description |
Descendents |
Methods (details)

toString (line 32)

Serializes a object into a string

- access: public

string

toString
()

---

Redefined in descendants as:

- DbModel::toString()
  : Short dump of the DbModel.
- MemModel::toString()
  : Short Dump of the MemModel.
- InfModelB::toString()
  : Short Dump of the InfModelB.
- InfModelF::toString()
  : Short Dump of the InfModelF.
- Literal::toString()
  : Dumps literal.
- Resource::toString()
  : Dumps resource.
- BlankNode::toString()
  : Dumps bNode.
- Statement::toString()
  : Dumps the triple.

Documentation generated on Thu, 7 Jul 2005 13:42:18 +0200 by phpDocumentor 1.3.0RC3
